# Using a Virtual Simulation Workshop to Teach Interns Evidence-Based Feedback Techniques

**DOI:** 10.7759/cureus.49709

**Published:** 2023-11-30

**Authors:** Dotun Ogunyemi, Birpartap S Thind, Kelly Chang, Sumayya Mohammed, Mariamu Osumah, Roberto Flores, Tommy Lee, Lisa Herring Sovory, Sarkis Arabian, Niren Raval

**Affiliations:** 1 Graduate Medical Education, Arrowhead Regional Medical Center, Colton, USA; 2 Medical Education, California University of Science and Medicine, Colton, USA; 3 Neurology, Arrowhead Regional Medical Center, Colton, USA; 4 Medical Education, University of California Riverside School of Medicine, Riverside, USA; 5 General Surgery, Arrowhead Regional Medical Center, Colton, USA; 6 Neurology, California University of Science and Medicine, Colton, USA; 7 Critical Care, Arrowhead Regional Medical Center, Colton, USA; 8 Family Medicine, California University of Science and Medicine, Colton, USA

**Keywords:** medical education, interns, osce, one-minute preceptor model, virtual simulation, feedback

## Abstract

Background

The Accreditation Council for Graduate Medical Education requires residents to demonstrate competence in integrating feedback into their daily practice. With the shift to virtual medical education during the pandemic, the need for new skills in delivering effective feedback through virtual media has emerged.

Methodology

This study aimed to assess the feasibility of a virtual bootcamp for interns, utilizing virtual simulation workshops to teach effective feedback skills. The curriculum employed a situated learning-guided participation framework. Virtual standardized students participated, and interns engaged in activities such as providing virtual feedback, completing self-assessments, and receiving instruction on feedback principles, including the one-minute preceptor’s five micro-skills. Interns repeated the feedback process, with virtual students providing assessments. Data were collected from 105 incoming interns at Arrowhead Regional Medical Center in June 2021 and June 2022, using Zoom® as the online platform.

Results

Competency assessments revealed a significant post-training increase in proficiency/expert milestones (88% versus 47%, p = 0.007). Interns’ self-assessments also significantly improved (18.02 versus 16.74, p = 0.001), particularly for previously trained interns (18.27 versus 16.7). Non-primary care interns outperformed primary care interns in milestone scores. The majority of interns (80%) reported valuable learning experiences during the workshop, with 70% expressing confidence in using the one-minute preceptor technique during residency. The one-minute preceptor step “reinforce what was right” was deemed the easiest, while “obtain commitment” and “explore emotional reaction” presented significant challenges.

Conclusions

This study demonstrates the potential of virtual workshops to enhance intern competency in delivering effective feedback through formal processes and the one-minute preceptor. These virtual approaches offer innovative alternatives to in-person teaching, enabling evaluation at higher levels of Miller’s pyramid of clinical competence.

## Introduction

The Accreditation Council of Graduate Medical Education (ACGME) requires that residents demonstrate interpersonal and communication skills that result in effective exchange of information and collaboration with patients, their families, and health professionals. Feedback is a process of communication that provides a constructive, objective appraisal of performance with the intention to improve skills [1]. The transition to a competency-based medical education framework has at its core the provision of adequate and multi-level assessment and feedback [2].

An effective feedback format includes confirming a trainee’s goals and needs, obtaining learners’ self-assessment, giving specific feedback on performance based on direct observation that includes both positive and constructive feedback, exploring the emotional reaction of learners, asking for understanding, problem-solving and developing an action plan, and establishing follow-up [3-5].

The one-minute preceptor model (OMP) is an evidence-based model to provide effective feedback, and it includes the following five key steps: get a commitment, probe for evidence, teach a general rule, reinforce what was done well, and correct mistakes [6,7]. Brief training in OMP has shown that residents’ confidence and teaching effectiveness improved after brief training in OMP [6]. The COVID-19 pandemic posed a challenge by limiting in-person instruction, requiring the medical community to increase virtual conferencing for medical education. Teaching effective feedback skills through virtual media is a new skill with sparse data regarding feasibility and efficacy [8]. Despite the key role of feedback in learning, studies have found that learners feel they receive inadequate feedback during clinical rotations [4].

The objectives of this study were to (1) design a virtual workshop to meet the ACGME requirement of teaching interpersonal and communication skills; (2) determine the feasibility of a virtual bootcamp for interns using a virtual simulation workshop to teach giving effective feedback to standardized students; (3) obtain baseline assessments of the interns on their competency in effective feedback skills; and (4) assess if the virtual workshop can increase the interns’ competency in using a formal feedback process and the OMP’s five micro-skills.

## Materials and methods

This was a prospective data collection of educational virtual bootcamps on effective feedback given to all incoming interns at the Arrowhead Regional Medical Center in June 2021 and June 2022. The conceptual framework utilized was situated learning-guided participation in which didactic and interactive activities facilitate independent learning [9]. The online delivery platform employed was Zoom®, given its accessibility, functionality, and ability to simulate the rotational format of Objective Structured Clinical Examination (OSCE) stations with breakout rooms. The virtual workshop utilized the OSCE with assessment at the “shows how” level of Miller’s (1990) pyramid. The virtual workshop was designed to meet the ACGME requirement of teaching interpersonal and communication skills.

Medical students were recruited and trained to play the role of virtual standardized students. The medical students were recruited by sending an email to all medical students. Interested medical students who responded were invited to attend a virtual training session which was one hour long. Medical students who participated were motivated by being involved in medical innovation and helping to train interns, which is perceived as a major incentive. Clinical scenarios for the standardized students, feedback scripts, a rubric checklist, a self-assessment tool, and an informative PowerPoint presentation were created by the lead author.

A 90-minute virtual workshop was conducted by the lead author who is a medical education expert. The workshop included a pre-intervention assessment, intervention on feedback skills, and post-intervention assessment. In the workshop, interns (1) gave feedback to the standardized student (acting as clinical students in the scenarios) based on clinical scenarios presented by the virtual standardized student using scripts and were assessed and graded by virtual students using a feedback checklist; (2) completed an online self-assessment on Poll Everywhere; (3) were taught effective feedback principles using a formal feedback tool based on ADAPT (Ask-Discuss-Ask-Plan Together) framework and the OMP’s five micro-skills [5,7]; (4) gave feedback to the virtual standardized students a second time using other provided scripts and were again assessed and graded by virtual students; and (5) completed the self-assessment tool.

Informed consent was waived by the institutional review board (protocol #20-45) as this was a prospective collection of educational data. The collected data were anonymized. Participation was voluntary and had no impact on the learners’ standing in their educational program. None of the investigators had any conflict of interest, and there was no extra funding, with investigators donating their time and expertise.

Statistical analysis was performed as indicated using SPSS version 21.0 (IBM Corp., Armonk, NY, USA). Statistical analysis included descriptive analysis, Student’s t-test, and chi-square test. A two-sided p-value <0.05 was accepted as significant. The observed feedback assessment scores graded by the standardized students were analyzed and the interns’ competency in giving effective feedback was categorized using ACGME milestones as novice, advanced beginner, competent, proficient, and expert [10]. Interns from internal medicine and family medicine were categorized as primary care while interns from general surgery, obstetrics and gynecology, emergency medicine, and psychiatry were categorized as non-primary care. Outcomes of the self-assessment and effective feedback assessments were compared by pre versus post-intervention, as well as by the presence or absence of previous training. Other factors analyzed included gender, ethnicity, and country of birth. These factors have been associated with negative or adverse academic outcomes, which have been attributed to implicit bias, biased assessments, stereotype threat, and cultural differences, related to a Westernized or dominant culture [11].

## Results

There were a total of 121 incoming interns who participated in the orientation bootcamps. Of the 121 interns, 59 participated during June 2021 and 62 participated during the June 2022 bootcamp. Of the total incoming interns, 105 (86.8%) completed associated assessment surveys. Of the 96 interns who provided demographic data, approximately 57% identified as Asian, 28% as non-Hispanic white, 9% as Latinx, and 6% as African American, with 52% males and 48% females. About 34% of the interns were born outside the United States, 60% were in primary care, and about 57% had previous training in feedback as part of the medical school curriculum (Table 1).

**Table 1:** Demographics of interns who participated in the virtual workshop (n = 105).

Category	Number	Percentage
Race		
Black	6	6.4
Latinx	8	8.5
Asian	53	56.4
White	27	28.7
Total	94	100
Gender		
Female	46	48
Male	50	52
Total	96	100
Birthplace		
Non-US born	28	35.4
US born	51	64.6
Total	74	100.0
Specialty		
Emergency Medicine	8	8.7
Family Medicine	26	28.3
Surgery	11	12.0
Internal Medicine	29	31.5
OBGYN	6	6.5
Psychiatry	12	13.0
Total	92	100
Previous training		
No previous training	48	57.1
Previous training	36	42.9
Total	84	100

Among the interns, “reinforce what was right” (n = 35%) was the most commonly recalled step in the OMP. Conversely, “obtain commitment” (n = 29%) proved to be the most challenging to remember. For the formal feedback, “giving positive feedback” (n = 42%) emerged as the most easily recalled step; conversely, “exploring emotional reaction” (n = 52%) was the most challenging for the interns to remember. Approximately 80% perceived that they learned a lot from this program, while 70% stated that they would be comfortable using OMP in the future during residency (Table 2).

**Table 2:** Interns’ self-assessments and perceptions regarding the virtual feedback training.

Self-assessment	Number	Percentage
I feel comfortable using the one-minute preceptor with other learners in residency (n = 79)	56	70.9
I learned a lot from this workshop about giving feedback (n = 80)	64	80
The step of the one-minute preceptor that was the easiest to remember (n = 79)		
Obtain a commitment (what)	10	12.7
Probe for supporting evidence (why)	9	11.4
Teach general rule	21	26.6
Reinforce what was right	28	35.4
Correct mistake	11	13.9
The step of the one-minute preceptor that was the most difficult to remember (n = 79)		
Obtain commitment (what)	23	29.1
Probe for supporting evidence (why)	16	20.3
Teach general rule	18	22.8
Reinforce what was right	12	15.2
Correct mistake	10	12.7
The step of the feedback format that was the most difficult to remember (n = 79)		
Positive feedback	3	3.8
Obtain learners’ self-assessment	14	17.7
Explore emotional reactions	41	51.9
Constructive feedback	9	11.4
Action plan/problem-solving	12	15.2
The step of the feedback format that was the easiest to remember (n = 80)		
Positive feedback	33	41.2
Obtain learners’ self-assessment	13	16.2
Explore emotional reaction	10	10
Constructive feedback	20	20
Action plan/problem-solving	4	5.0

Both the interns’ self-assessment scores and the observed assessment scores given by standardized students significantly increased after training. Interns whose competency was graded as reaching proficiency or expert milestones significantly increased from pre to post-training assessment (p = 0.017). Interns who reported previous training on feedback in the medical school reported increased use of a formal process for giving feedback and had significantly increased self-assessment scores (p = 0.14). Interns from non-primary care specialties compared to those from primary care specialties had significantly higher observed assessment scores and higher milestones (p = 0.009 and p = 0.003, respectively). The observed assessment scores were also significantly increased for interns identifying as non-Hispanic whites compared to those of other ethnicities (12.35 vs. 12.29, respectively, p = 0.026). The only significant difference based on gender was that male interns found positive feedback the easiest step to remember in the formal feedback process, whereas female interns found constructive feedback the easiest to remember (p = 0.023) (Table 3).

**Table 3:** Significant differences in virtual simulation educational outcomes for interns from comparisons of pre- versus post-training, previous training, specialty, gender, and ethnicity. Standard deviation: represented in parenthesis (). Primary care: Internal Medicine and Family Medicine interns. Non-primary care: General Surgery, Emergency Medicine, Psychiatry, and Obstetrics and Gynecology interns. Non-white: Asian, Hispanic, and African American interns. * = variables where pre and post-values were combined during the analysis. The numbers are different due to incomplete survey responses. Specifically, the standardized students only submitted 32 completed rubric forms on interns categorized as primary care interns, and 22 categorized as non-primary care. However, of the interns themselves, 42 interns categorized as primary care submitted the one-minute preceptor survey, and 30 interns categorized as non-primary care interns submitted the one-minute preceptor survey.

Variables	Pre-training (n = 82)	Post-training (n = 82)	P-value
Mean self-assessment score	2.85 (0.44)	3.09 (0.46)	0.004
Self-assessment score	16.74 (2.80)	18.02 (2.74)	0.001
Milestones	(n = 39)	(n = 20)	0.017
Novice	2 (5.1%)	0	
Advanced beginner	8 (20.5%)	0	
Competent	11 (28.2%)	3 (15%)	
Proficient	17 (43.6%)	13 (65%)	
Expert	1 (2.6%)	4 (20%)	
Mean milestone score	3.18 (0.97)	4.05 (0.61)	0.001
Observed score by standardized student	11.21 (3.22)	14.05 (1.61)	<0.001
Variables	No previous training (n = 92)*	Previous training (n = 66)*	P-value
I followed a formal process for giving feedback for the clinical case	44 (48.4%)	45 (69.2%)	0.014
Total self-assessment score	16.71 (2.95)	18.27 (2.45)	<0.001
Variables	Primary care (n = 32)	Non-primary care (n = 22)	P-value
Observed score by standardized student	11.47 (3.17)	13.50 (2.30)	0.009
Mean milestone score	3.22 (0.91)	3.95 (0.79)	0.003
Milestones			P-value
Novice	1 (4.5%)	0 (0.0%)	0.027
Advanced beginner	7 (21.9%)	1 (4.5%)	
Competent	8 (25.0%)	4 (18.2%)	
Proficient	16 (50.0%)	12 (54.5%)	
Expert	0 (0%)	5 (22.7%)	
The step of the one-minute preceptor that was the most difficult to remember	Primary care (n = 42)	Non-primary care (n = 30)	P-value
Teach general rule	11(26.2%)	6 (20.0%)	0.02
Reinforce what was right	10 (23.8%)	2 (6.7%)	
Probe for supporting evidence (why)	9 (21.4%)	7(23.3%)	
Obtain commitment (what)	5 (11.9%)	13 (43.3%)	
Correct mistake	7 (16.7%)	2 (6.7%)	
The step of the feedback format that was the easiest to remember	Female (n = 41)	Male (n = 31)	P-value
Positive feedback	21 (51.2%)	10 (30.3%)	0.023
Obtain learners’ self-assessment	8 (19.5%)	4 (12.1%)	
Explore emotional reaction	2 (4.9%)	8 (24.2%)	
Constructive feedback	10 (24.4%)	8 (24.2%)	
Action plan/problem-solving	0 (0.0%)	3 (9.1%)	
Variables	Non-white (n = 113)*	White (n = 38)*	P-value
Observed score by standardized student	12.29 (3.0)	12.35 (3.06)	0.026

## Discussion

We designed and implemented a virtual synchronous simulation workshop using medical students as virtual standardized students to assess and facilitate the teaching of incoming interns on effective feedback using a standardized format and the OMP. Our results showed that from pre- to post-training, there were significant increases both in interns’ self-assessment of efficacy in giving feedback and interns’ competency at the proficient or expert level which increased from 46.2% to 85%, as assessed by the standardized students.

Studies on virtual synchronous clinical assessments and OSCE-themed formats are sparse with the few published reports mainly descriptive, short reports or evaluations of student participants [12]. A recent publication described the transition of the ACGME faculty development hub at Michigan State University College of Osteopathic Medicine Statewide Campus System (MSUCOM SCS) from a traditional in-person educational course to a virtual format using Zoom as highly effective and completely or mostly met all course learning objectives [13]. A short report on 26 residents revealed that the virtual format was moderately reliable for assessing key obesity competencies among residents [14]. Educators agree that effective feedback procedures are fundamental to training and competency-based education. Hence, our study adds to the literature by demonstrating that virtual OSCE-based assessment of feedback for incoming interns over two consecutive years was feasible and effective. The pandemic is driving a paradigm shift in GME for both education programming and staffing needs; these changes may likely persist, as restrictions to in-person learning are lifted [13]. Furthermore, the exponential growth of telehealth with the pandemic requires that physicians develop skills for utilizing a virtual platform successfully; thus, incorporating a virtual educational format into residency training is important.

Thampy et al. noted that as OSCE stations are designed to reflect everyday clinical competencies, online formats must meet four themes, namely, (a) optimizing assessment design for online delivery, (b) ensuring clinical authenticity, (c) recognizing and addressing feelings and apprehensions, and (d) anticipating challenges through incident planning and risk mitigation. We addressed these themes by using Zoom®; we trained medical students to ensure clinical authenticity; detailed orientation, explanations, and icebreakers addressed expectations and decreased anxiety; and no incidents occurred because we conducted ‘‘trial’’ presentations of content and ensured robust technology support [15].

Our study also revealed other factors that may contribute to the interns’ competency in giving or recognizing effective feedback. Interns who reported previous training on feedback in medical school had significantly higher self-assessment scores even though there were no significant differences between competency assessed by standardized students. However, studies have shown physicians are poor at self-assessment in the absence of external guidance and have emphasized that feedback is essential for deliberate practice and competency-based training [16].

One advantage of virtual educational programming is averting the risk of contagious infection in view of the recent pandemic. This also saves the requirement of a large physical space that will hold all the interns, standardized students, and administrators. Furthermore, it also decreases the human administrative and coordination requirement for an in-person event. It is more likely in an in-person event that an incentive is required for standardized students and saves on the cost of providing lunch for all the attendees.

Interns in non-primary care specialties had significantly higher observed assessment scores and higher milestones than those from primary care specialties. This may be a spurious finding requiring future well-designed studies.

Assessment scores by standardized students were significantly higher for interns identifying as non-Hispanic whites compared to other ethnicities which is consistent with the literature [11]. Wijesekera et al. reported that black students received consistently lower grades than non-black students across all clerkships and were also roughly two-thirds less likely to be inducted into AOA and concluded that the findings suggest the possibility of biased selection [17]. The importance of addressing biased assessments has been recognized; Washington University School of Medicine developed the Commission for Equity in Clinical Grading to understand and address bias in clerkship grading and AOA nomination [18].

It is important to note that the step of the OMP that interns found the easiest to remember was “reinforce what was right” and what they found the most difficult to remember was “obtain commitment.” For the formal feedback format, “giving positive feedback” was the step the easiest to remember, and “exploring emotional reaction” was the most difficult to remember. This information is practical and useful and can be used for teaching and curriculum development.

Limitations

Our study limitations included a small sample size and incomplete data, possibly leading to a type 2 error. However, we did obtain statistically significant results. The online self-assessment surveys were voluntary and were completed by the majority of interns (85%). Interns were observed and graded by standardized students using a hardcopy form which created challenges with timely completion and collection. A limitation of this study is the self-assessment by the interns, which could be subjective and therefore prone to bias. Furthermore, the medical students may also have been hesitant and favorably biased in assessing their senior colleagues. Moreover, no inter-rater reliability tests were done. We only obtained immediate short-term assessments with no longitudinal follow-up to assess long-term skill retention. Future longitudinal studies are required. Our findings may not be generalizable to other learning environments, as our study was done in a county hospital in southern California that has a mid-sized GME. Furthermore, we used a convenience cohort with no controls and no randomizations.

## Conclusions

In summary, a virtual workshop can assess and improve the competency of interns in giving effective feedback by using a formal process and the OMP. A virtual simulation workshop is feasible and requires fewer resources than an in-person workshop. Medical students are enthusiastic, ready to help, and easy to train as standardized students or patients for residents’ simulation workshops. These interactions can facilitate bonding and engagement between residents and medical students. GME and program directors should consider including virtual simulation training on feedback as part of the orientation curriculum for incoming interns. Future studies could develop a longitudinal curriculum with multiple assessments over time, provide online formats for the observed assessments by the standardized students, and include multiple sites.
